# Fatal posterior circulation stroke with persistent hiccups, sinus arrest and post-hiccup syncope: A case report

**DOI:** 10.1097/MD.0000000000033053

**Published:** 2023-02-17

**Authors:** Na Zhang, Hao Liang, Xibing Wang, Hong Wang

**Affiliations:** a Department of Cardiology, Affiliated Hospital of Chengde Medical University, Chengde, Hebei Province, China.

**Keywords:** case report, persistent hiccups, posterior circulation stroke, post-hiccup syncope, sinus arrest

## Abstract

Diagnosis of posterior circulation stoke is difficult, and magnetic resonance imaging especially diffusion-weighted imaging is superior to computed tomography. Persistent hiccups, sinus arrest, and post-hiccup syncope are extremely rare symptoms of posterior circulation stroke. However, there is no effective treatment for persistent hiccup.

**Patient concern and diagnosis:**

We describe a case of a 58-year-old hypertensive woman diagnosed with acute posterior circulation stroke who presented with persistent hiccups, sinus arrest, and post-hiccup syncope. Diffusion-weighted imaging revealed a high-intensity signal involving the left middle cerebellar peduncle and several spotted areas in the right occipital lobe.

**Interventions:**

Permanent pacemaker was implanted and metoclopramide was used to treat persistent hiccups.

**Outcome:**

The patient developed aspiration pneumonia and morbid dysphoria, and eventually died.

**Lessons:**

Posterior circulation stroke can cause cardiovascular and respiratory dysfunction. Consequently, physicians should pay more attention to posterior circulation lesions in patients with arrhythmia and syncope. An effective method to treat persistent hiccups is urgently needed.

## 1. Introduction

The middle cerebellar peduncles are the largest system connecting the cerebellum to the pons.^[1,2]^ The incidence of isolated middle cerebellar peduncles infarction is rare, accounting for approximately 0.12% of acute strokes.^[3]^ The common symptoms include vertigo, ataxia, hearing loss, facial paralysis and Horner syndrome.^[1,4–7]^ Patients with occipital stroke mainly show cortical blindness, unilateral vision loss and occipital headache.^[8]^ Both regions are supplied by the posterior circulation. In this study, we present a case of posterior circulation infarction located in the above 2 regions. However, in addition to the above-mentioned common symptoms, the patient also experienced persistent hiccup, sinus arrest and post-hiccup syncope, which was a rare phenomenon.

## 2. Patient information

**Figure 1.**
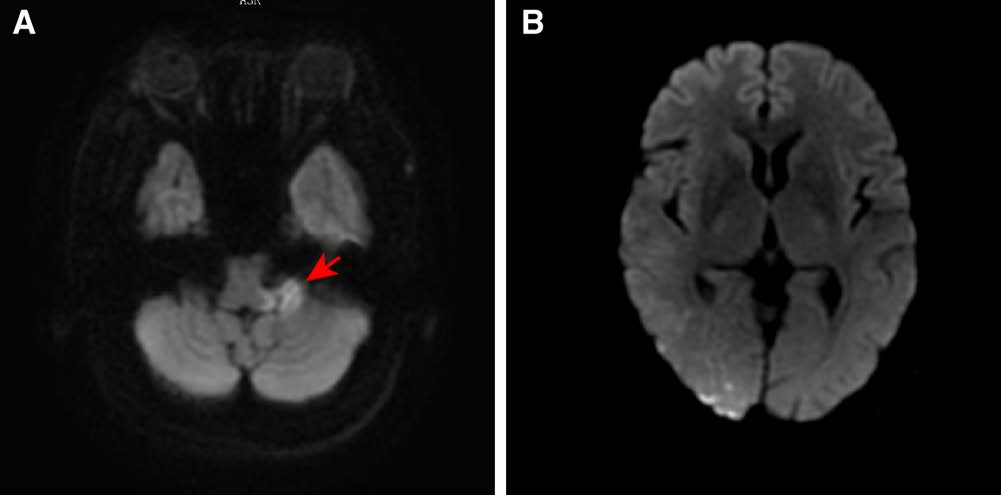
Diffusion-weighted imaging (DWI) in the brain. (A) A high-intensity signal involving the left middle cerebellar peduncle; (B) several spotted areas in the right occipital lobe.

**Figure 2.**
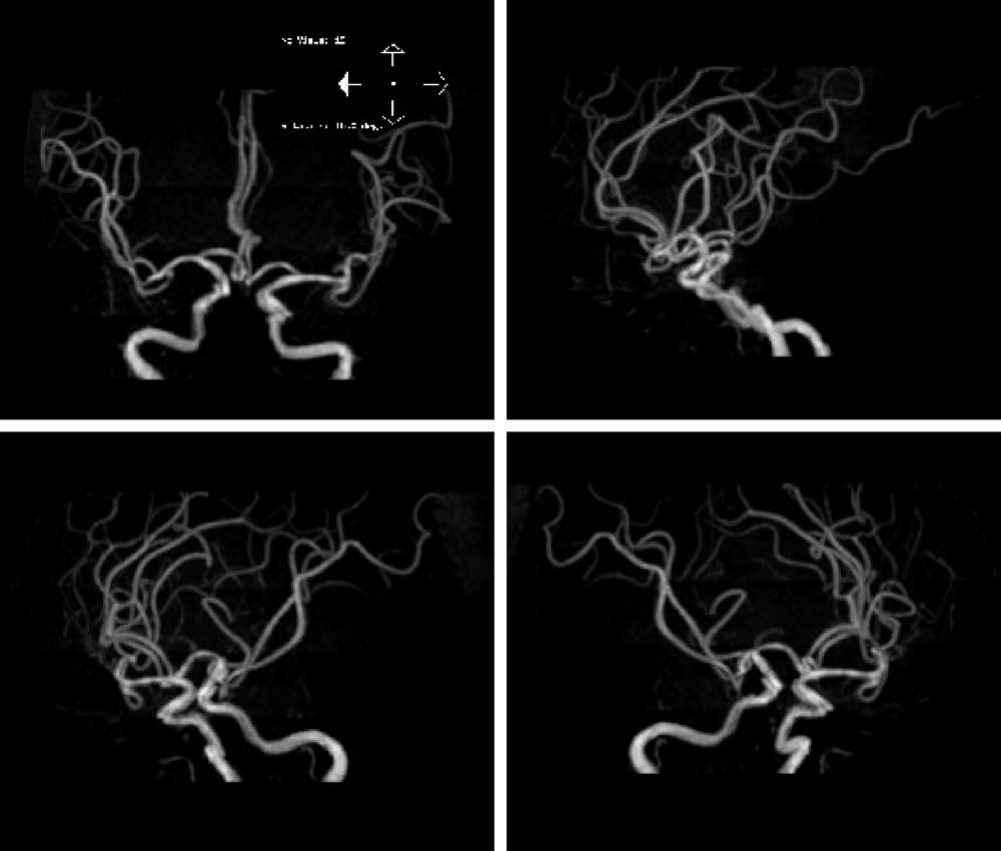
Magnetic resonance angiography (MRA) suggested atherosclerosis, and no severe stenosis or occlusion was found.

A 58-year-old Chinese woman presented to the cardiology ward of our hospital with a 10-day history of vertigo, nausea, vomiting, occipital throbbing headaches, ataxia and a 6-day history of hiccups, sinus arrest and post-hiccup syncope. Ten days before admission, the patient experienced vertigo, nausea, vomiting, occipital throbbing headaches, tinnitus, hearing loss and ataxia, and the patient did not have any sensory deficits, limb dysmetria, language deficits or dysphagia. The patient went to the outpatient department for help, and the outpatient department failed to make a definite diagnosis. No significant abnormal results were found in abdominal ultrasound, gastroscopy, trans-cranial Doppler, carotid artery ultrasonography, vertebral artery ultrasonography, and a noncontrast head computed tomography (CT) scan. Six days before admission, the patient had syncope that was relieved for approximately 5 seconds. Five days before admission, the patient presented to the neurology ward of a military hospital. Diffusion-weighted imaging revealed a high-intensity signal involving the left middle cerebellar peduncle and several lacunar regions in the right occipital lobe (Fig. 1), which was consistent with the diagnosis of acute posterior circulation stroke. Magnetic resonance angiography suggested atherosclerosis, and no severe stenosis or occlusion was found (Fig. 2). Since then, the patient had recurrent episodes of hiccups and post-hiccup syncope every day. After recovery of consciousness, the patient had shortness of breath, which could be relieved in a few minutes. The electrocardiogram and blood pressure monitor exhibited sinus arrest and lowered blood pressure when the patient had the hiccups and syncope. The frequency of the patient hiccups and cough syncope gradually increased. All these factors led to the occurrence of depression, irritability and fear, affecting the patient diet and sleep. Therefore, the patient condition worsened each day. To ensure the safety of the patient life, the physicians placed a temporary pacemaker 3 days before admission. However, the patient hiccups still occurred several times a day, and they were accompanied by dysphoria but not syncope. Unfortunately, the patient developed fever, cough and expectoration 1 day before admission. To evaluate the causative diseases of arrhythmia and syncope, the patient was admitted to the cardiology ward of our hospital.

## 3. Clinical findings

Thyroid function, kidney function, hepatic organ function, electrolytes, brain natriuretic peptide, and troponin were normal. Baseline electrocardiogram showed normal sinus rhythm with a heart rate that fluctuated between 60 beat per minute (bpm) to 80 bpm and there were no significant changes in the ST segments or T waves. A neurological examination upon admission revealed hearing loss, facial paralysis and ataxia. On the day of admission, a pulmonary CT scan showed pneumonia, which was consistent with the diagnosis of aspiration pneumonia. After admission, the 24-hour dynamic electrocardiogram results were as follows: sinus rhythm dominated, intermittent pacing rhythm, the heart rate fluctuated from 60 bpm to 85 bpm, the average heart rate was 71 bpm, 139 atrial premature beats, and no ventricular arrhythmia.

## 4. Therapeutic intervention

The patient was treated for stroke with drugs. Antibiotics were used to treat the aspiration pneumonia. The patient had recurrent hiccups. We asked the Department of Neurology for consultation many times, but there was no good treatment for the patient persistent hiccups. Metoclopramide was used intermittently to relieve the hiccups. One day after admission, the BP of the patient dropped to 10.4/6.67 kPa, and dopamine was used to improve blood pressure. Three days after admission, the hiccups were still recurring, accompanied by a pacing rhythm, so we replaced the temporary pacemaker with a permanent pacemaker.

## 5. Outcomes and follow-up

Five days after admission, the patient developed fever again, with impotent expectoration, glossocoma and dyspnea. Therefore, we added moxifloxacin combined with cefoperazone-sulbactam to treat the patient pneumonia. However, the patient pulmonary infection could not be controlled. Nine days after admission, the patient was forced to undergo endotracheal intubation and to be placed on a ventilator to assist ventilation due to the aggravation of dyspnea. Ten days after admission, we found that the patient had no cough reflex during sputum aspiration. Due to the poor condition of the patient and the implantation of a permanent pacemaker, magnetic resonance imaging (MRI) could not be used to assess whether the patient had new infarct lesions. Therefore, we recommended a tracheotomy, but the patient family refused, gave up the treatment and took the patient home. A few days after returning home, the patient died. The patient did not undergo autopsy because the family did not allow.

## 6. Discussion

Making the diagnosis of posterior circulation stroke is a challenge for physicians because the clinical manifestations are complex and subtle and can vary.^[9]^ Approximately 37% of posterior circulation strokes are misdiagnosed in the emergency department, which is more than 3 times the misdiagnosis rate of anterior circulation strokes.^[10]^ The main manifestations of patients with posterior circulation strokes are vertigo, nausea and vomiting. Therefore, this patient was suspected to have gastrointestinal diseases, but no serious abnormalities were found on abdominal ultrasound and gastroscopy. The patient was also suspected to have posterior circulation disease because of vertigo and ataxia, but no new lesions were found on head CT. Ultimately, she missed the best opportunity for acute interventions and improved outcomes. This also confirms that noncontrast head CT is limited for the detection of posterior circulation stroke and that brain MRI is more accurate than CT for diagnosing this stroke.^[11]^ The patient condition was progressing continuously, but due to the implantation of a pacemaker, it was impossible to evaluate whether there was a new infarct focus with MRI.

Hiccups are a universal phenomenon. Most hiccups are transient and self-limited and can occur in healthy people and children. Persistent hiccups, which are hiccups lasting for more than 48 hours but < 1 month,^[12]^ are rare and may indicate the presence of an underlying pathological process. Some scholars believe that the reflex arc of the hiccup is as follows: The afferent pathway of hiccups includes the sensory fibers of the vagal nerve, the phrenic nerve, and sympathetic nerves from Th6 to Th12;^[12,13]^; The hiccup centers mainly include the medulla oblongata, the periaqueductal gray, subthalamic nuclei, phrenic nerve nuclei, reticular formation, and C3 to 5 cervical cord;^[12–14]^ the hypothalamus and temporal lobe may have a greater influence on the arc; and; The efferent pathway of hiccups includes the phrenic nerves to the diaphragm, the external intercostal nerves (Th1–Th11) to the intercostal muscles, the cervical spinal nerves to the anterior scalene muscles, and the recurrent laryngeal portion of the vagal nerve to the glottis, which causes glottal closure.^[12,14]^

Many diseases, such as stroke,^[15,16]^ cancer,^[17]^ surgery,^[18,19]^ and COVID-19,^[20]^ can cause hiccup, which can be divided into the following 4 categories according to the etiologies: central, peripheral, global and medication-derived causes.^[21]^ The incidence rate of hiccups in ordinary inpatients is 0.054%,^[22]^ in cancer patients is 3.9% to 4.5%,^[23]^ and in stroke patients is approximately 0.3% (16 per 5309).^[24]^ The most responsible lesion for producing hiccup was the tegmentum of the medulla oblongata. If the stimuli are constant and have sufficient magnitude, persistent hiccups will occur.^[25]^ This patient had persistent hiccups due to infarction of the left middle cerebellar peduncle and the right occipital lobe, which may also be involved in the formation of a hiccup reflex arc. Persistent hiccups affect the patient ability to eat and interact socially and can cause severe debilitation, dehydration, insomnia, protracted mental stress, and even death.^[26]^ It is very unfortunate that all these conditions occurred in this patient.

Many studies have found that a variety of methods can treat persistent hiccup, such as behavioral modifications, physical maneuvers, pharmacologic and nonpharmacologic interventions.^[21]^ The common pharmacologic interventions include amitriptyline, baclofen, metoclopramide, and valproic acid.^[27]^ However, all drugs have certain adverse effects, and the US Food and Drug Administration only approves chlorpromazine to treat hiccups. However, all these evidences are limited and insufficient, and most of comes them come from case reports and noncontrolled retrospective studies. nonpharmacologic interventions include acupuncture, ^[28,29]^ positive pressure ventilation, transcutaneous phrenic and vagal nerve stimulation, ultrasound-guided continuous phrenic nerve block, anterior and posterior diaphragm kinesio taping,^[30]^ and so on. However, most of these come from case reports, and only acupuncture treatment for hiccup comes from systematic reviews, which show that the evidence is not sufficient. To date, there are no unified guidelines to guide the diagnosis and treatment of persistent hiccup. In this case, we used metoclopramide to treat the hiccups but failed. Due to the obvious dysphoria of the patient, acupuncture treatment was not performed.

In this patient, post-hiccup syncope occurred after a hiccup episode of 5 to 10 seconds. The pathogenesis is similar between post-hiccup syncope and cough syncope.^[31]^ When cough or hiccups occur, the rise in intrathoracic pressure acutely lowers the venous return and cardiac output, leading to a decrease in cerebral perfusion and loss of consciousness.^[32]^ Baroreflex activation leads to a vagal response, causing vasodilation, bradycardia,^[33]^ and even sinus arrest. This patient had a series of hiccups, post-hiccup syncope, sinus arrest, and lowering of her BP when diffusion-weighted imaging revealed acute infarction of the left middle cerebellar peduncle and the right occipital lobe. Similar reports have been described before, but most of the lesions are in the medulla oblongata.^[34]^ These regions may have some relationship with the vasomotor and autonomic centers of the medulla oblongata.

It is very rare that persistent hiccup, sinus arrest and syncope occur at the same time after stroke. The patient condition progressed rapidly after hiccup attack. There was no effective treatment for hiccup, and syncope occurred repeatedly, so a pacemaker was placed after reviewing some case reports.^[35]^ After that, the patient still had hiccups but without syncope. Unfortunately, the patient eventually died because of aspiration pneumonia and the negative effects of persistent hiccups. We urgently need an effective treatment for hiccups.

## 7. Conclusion

Diagnosis of posterior circulation stroke is difficult, and MRI is more sensitive and accurate than CT. The infarction of the left middle cerebellar peduncle and the right occipital lobe can cause persistent hiccups, post-hiccup syncope and sinus arrest, and all these symptoms aggravate the patient condition and cause death. These findings showed that posterior circulation stroke can cause cardiovascular and respiratory dysfunction. Consequently, physicians should pay more attention to posterior circulation lesions in patients with arrhythmia and syncope.

At present, there are no unified guidelines to guide the diagnosis and treatment of persistent hiccup. When a patient has a stroke with persistent hiccup, we should try to treat the patient in a variety of ways to improve the prognosis of the patient. It is expected that a prospective, multicenter, large sample randomized controlled study on persistent hiccup will be conducted to provide an effective evidence-based medical basis for the diagnosis and treatment of persistent hiccup.

## Acknowledgments

The authors thank the patient and her family for their participation.

## Author contributions

**Supervision:** Hao Liang, Xibing Wang.

**Writing – original draft:** Na Zhang.

**Writing – review & editing:** Hong Wang.
